# Assessing the public health risk from ESKAPE pathogens including MRSA and VRE on high-touch shopping cart surfaces

**DOI:** 10.3934/publichealth.2026012

**Published:** 2026-02-03

**Authors:** Ziad Jaradat, Batool Khataybeh, Qutaiba Ababneh, Anas Al Nabulsi, Ragad Musa, Lamice Shurafa, Nisrina Ali, Maram Zahrawi, Farah Harbieh, Jude Nassar, Abeer Idrees, Bara'ah Eyadat, Ekhlas Al-Rousan

**Affiliations:** 1 Department of Biotechnology and Genetic Engineering, Jordan University of Science and Technology, P. O Box, 3030, Irbid, Jordan; 2 Department of Nutrition and Food Technology, Jordan University of Science and Technology, P. O Box, 3030, Irbid, Jordan

**Keywords:** shopping carts, ESKAPE pathogens, environmental contamination, MRSA, VRE, community transmission

## Abstract

Increased antimicrobial resistance (AMR) poses a significant threat to global health. This study investigated the prevalence, antibiotic susceptibility, and biofilm-forming ability of ESKAPE pathogens on shopping cart handles in Jordan. Swab samples were collected from 820 shopping carts used in grocery stores, and ESKAPE bacteria were identified using microbiological and molecular methods. The most prevalent species isolated was *Staphylococcus aureus*, which was recovered from 4.8% of the samples, followed by *Acinetobacter baumannii* (2.1%), *Escherichia coli* (2%), and *Enterococcus spp*. (0.73%). Most isolates were sensitive to antibiotics, though two *E. faecium* isolates showed resistance to vancomycin (VRE), and 17 *S. aureus* isolates were methicillin-resistant (MRSA). The ability of the isolates to form biofilm varied, with most *S. aureus* being non-formers (53.8%), whereas *A. baumannii* isolates were predominantly strong formers (53%). The presence of ESKAPE pathogens, including clinically significant strains like MRSA and VRE, on frequently touched shopping cart handles highlights the role played by these surfaces as potential reservoirs for transmission in the community.

## 1. Introduction

The term ESKAPE is an acronym for a group of nosocomial pathogens that are known for their antimicrobial resistance. ESKAPE stands for *Enterococcus* spp., *Staphylococcus aureus*, *Klebsiella pneumoniae*, *Acinetobacter baumannii*, *Pseudomonas aeruginosa*, and *Escherichia coli* [1]. Generally, *Enterococcus spp*., *S. aureus*, *K. pneumoniae*, and *E. coli* are natural colonizers of humans and animals, being opportunistic pathogens [2]. *A. baumannii* and *P. aeruginosa* are found naturally in the environment, and both are recognized for their ability to acquire antimicrobial resistance by horizontal transfer [3]. The acronym ESKAPE includes opportunistic pathogens that can escape from antimicrobial actions through random genetic mutations or mobile genetic elements, or through horizontal gene transfer [2]. Indeed, resistance of ESKAPE to many antibiotics, including last resort, has been recorded.

Antimicrobial resistance (AMR) occurs when bacterial pathogens develop survival mechanisms upon exposure to antibiotics designed to eliminate them [4]. According to the World Health Organization (WHO), AMR is a global health concern that is categorized as the fifth most urgent threat to global health. WHO projects that in 2050, more than 10 million people will die due to AMR unless new antibiotics are discovered or new therapeutic protocols are developed [5]. Several factors cause the spread of antimicrobial resistance, including the abusive use of antimicrobial agents without the strict supervision of medical staff, excessive prescription of drugs by unauthorized personnel, and subsequent ineffective therapy [6]. The ability of ESKAPE pathogens to escape the effect of antimicrobial agents aids in causing most of the nosocomial infections, thus increasing the mortality rate worldwide [7]. Diseases caused by ESKAPE pathogens are alarming because effective and safe treatment options are limited due to their growing resistance and stubbornness [8].

ESKAPE pathogens mainly affect immunocompromised, elderly, and hospitalized patients, leading to hard-to-treat infections, increasing mortality and morbidity rates, as well as prolonging hospital stays [1],[9]. Several fatal diseases are mainly associated with infection by one of the ESKAPE pathogens, such as meningitis, wound infections, pneumonia, bacteremia, and urinary tract infections [10]. In fact, ESKAPE pathogens were responsible for more than 80% of global deaths due to AMR organisms in the year 2019 [11]. The WHO categorized these six pathogens into two groups based on the urgency to develop new antibiotics to treat their infections. Vancomycin and methicillin-resistant *S. aureus* and vancomycin-resistant *E. faecium* are in the high-priority group list, whereas the extended spectrum β-lactamase (ESBL) or carbapenem-resistant *K. pneumoniae* and *Enterobacter* spp. and carbapenem-resistant *P. aeruginosa* and *A. baumannii* are in the critical priority group list of pathogens [7].

ESKAPE pathogens use several mechanisms to resist the effect of antibiotics, including modification of antibiotic binding sites, reduced intracellular antibiotic accumulation by increasing efflux pumps of the antibiotic or by decreasing the membrane permeability, and antibiotic inactivation or alteration by an irreversible cleavage catalyzed by an enzyme [12]. They are also distinguished by their ability to form biofilms, which helps in protecting the pathogens from the immune cells, exchanging nutrients and resistance genes between the cells, and protecting persister cells that are tolerant to antibiotics [13]. In addition, ESKAPE pathogens, with their capability to form strong biofilms, become hard to clean from hospitals as they are inaccessible to disinfectants such as alcohol, hydrogen peroxide, and chlorine-based formulations. They thus escape from being killed and might even develop resistance against some of these disinfectants [14].

Shopping cart handles are one of the most highly touched objects by people in malls, hypermarkets, and other facilities, being one of the leading sources of bacteria in public facilities [15]. Several studies have reported the isolation of different pathogenic and non-pathogenic bacteria from shopping cart handles [15]–[23]. The prevalence of ESKAPE pathogens in hospital settings has been studied extensively. However, studies on the prevalence and isolation of ESKAPE pathogens from the environment and, in particular, from the handles of shopping carts are scarce. Therefore, this research aimed to study the presence of ESKAPE bacteria on shopping cart handles in different cities in Jordan. Moreover, it aimed to investigate the antibiotic resistance and biofilm formation abilities of the obtained bacterial isolates.

## 2. Materials and methods

### 2.1. Sample collection

Between October 2021 and March 2023, 820 swab samples were collected from shopping cart handles in three major cities in Jordan, namely Amman, Irbid, and Zarqa. Sterile swabs were immersed in 3 mL of brain heart infusion (BHI) broth (Oxoid, UK) and used for surface sampling by moving the swab back and forth across the surface area of shopping carts' handles. Shopping carts from large supermarkets, in which the carts are heavily used, were chosen for the study. The supermarkets were chosen based on their location, such as malls where thousands of people shop every day. The collected swabs were maintained under aseptic conditions and transported to the laboratory within 2 h of sampling. Swabs were incubated at 37 °C for 24 h with a rotation speed of 100 rpm.

### 2.2. Isolation and molecular identification of ESKAPE bacteria

All enriched samples were streaked onto bile-esculin agar (Oxoid, UK), Baird-Parker agar (BPA) (Oxoid, UK), mannitol salt agar (MSA) (Oxoid, UK), eosin-methylene-blue (EMB) agar (Oxoid, UK), CHROMagar *Acinetobacter* agar (CHROMagar, France), and cetrimide agar (Oxoid, UK) to isolate presumptive colonies of *Enterococcus* spp., *Staphylococcus aureus*, *Klebsiella pneumoniae*, *Escherichia coli*, *Acinetobacter baumannii*, and *Pseudomonas aeruginosa*, respectively. The plates were incubated at 37 °C for 24–48 h under aerobic conditions. After incubation, all presumptive isolates were preserved at −80 °C for further analyses. Genomic DNA was extracted from the samples using the in-house phenol-chloroform method. Molecular identification of ESKAPE bacterial isolates was performed by PCR (Applied Biosystems, USA) using species-specific primer sets (IDT, USA), as listed in Table 1. The DNA of *E. faecalis* 29212, *E. faecium* BAA–2316, *S. aureus* 33591, *K. pneumonia* BAA–2146, *A. baumannii* 19606, *P. aeruginosa* BAA–2114, and *E. coli* BAA–2452 was used as a control in all PCR amplifications based on the identified bacterial type.

**Table 1.** Primers used in this study.

Bacteria	Primer name	Primer sequence (5′–3′)	Amplicon size (bp)	References
*E. faecalis*	ddl	F-ATC AAG TAC AGT TAG TCT R-ACG ATT CAA AGC TAA CTG	941	[24]
*E. faecium*	EM1	F-TTG AGG CAG ACC AGA TTG ACG R-TAT GAC AGC GAC TCC GAT TCC	658	
*S. aureus*	Nuc	F-GCG ATT GAT GGT GAT ACG GTT R-AGC CAAGCC TTG ACG AAC TAA AGC	279	[25]
MRSA	MecA	F-CAT TGA TCG CAA CGT TCA ATT T R-CGG TTT TAA AGT GGA ACG AAG GT	310	
*K. pneumoniae*	phoE	F-TGGCCCGCGCCCAGGGTTCGAAA R-GATGTCGTCATCGTTGATGCCGAG	368	[26]
*Acinetobacter* spp.	recA	F-CCTGAATCTTCTGGTAAAAC R-GTTTCTGGGCTGCCAAACATTAC	425	[27]
*A. baumannii* and *A. nosocomialis*	gyrB	F-CACGCCGTAAGAGTGCATTA R-AACGGAGCTTGTCAGGGTTA	294	
*A. baumannii*	ITS	F-CATTATCACGGTAATTAGTG R-AGAGCACTGTGCACTTAAG	208	
*A. pittii*	AGS3	F-CTCAAGAGTTTAGATTAAGCAAT R-GTCCGTGCGATTCTTCATCG	150	
*P. aeruginosa*	PA-SS	F-GGGGGATCTTCGGACCTCA R-TCCTTAGAGTGCCCACCCG	956	[28]
*E. coli*	UspA	F-CCGATACGCTGCCAATCAGT R-ACGCAGACCGTAGGCCAGAT	884	[29]
*E. coli* O157:H7	rfbE	F-CAGGTGAAGGTGGAATGGTTGTC R-TTAGAATTGAGACCATCCAATAAG	296	[30]

### 2.3. Antibiotic susceptibility testing

Antibiotic susceptibility testing was performed following the Kirby–Bauer disk diffusion and the minimal inhibitory concentration (MIC) method according to CLSI guidelines [31], using the antibiotics listed in Table 2. All antibiotic discs were purchased from Oxoid, UK. Bacterial strains were cultured on LB agar plates and incubated overnight at 37 °C. One to three colonies were then suspended in 1 mL of 0.9% sterile saline and adjusted to 0.5 McFarland at 625 nm. Mueller Hinton agar (Oxoid, UK) plates were inoculated with the test strains using the swab surface method, antibiotic discs were placed, and plates were incubated at 37 °C for 18–24 h. Inhibition zone diameters were measured using a ruler, and the isolates were classified as sensitive (S), intermediate (I), or resistant (R) based on the CLSI antibiotic susceptibility breakpoints.

The minimal inhibitory concentration (MIC) was performed in 96-well microtiter plates using the broth microdilution method [32]. For colistin sulfate, Polysorbate 80 (Santa Cruz Biotechnology, INC., USA) was added to each well at a final concentration of 0.002% [33].

**Table 2.** Antibiotics used in this study.

Antibiotics	Antibiotic family	*E. faecalis/E. faecium*	*S. aureus*	*K. pneumoniae*	*A. baumannii*	*E. coli*
Ampicillin (10 µg)	β-lactams			+		+
Ampicillin/sulbactam (10/10 µg)		+			+	+
Piperacillin (100 µg)					+	+
Piperacillin-tazobactam (100/10 µg)					+	
Cefepime (30 µg)	Cephalosporins			+	+	+
Ceftriaxone (30 µg)				+	+	+
Ceftazidime (30 µg)				+	+	+
Ceftaroline (30 µg)						+
Cefoxitin (Cayman, USA)			+			
Imipenem (10 µg)	Carbapenems			+	+	+
Meropenem (10 µg)				+	+	+
Doripenem (10 µg)					+	
Ciprofloxacin (5 µg)	Fluoroquinolones	+	+	+	+	+
Levofloxacin (5 µg)		+			+	
Norfloxacin (10 µg)		+				
Nalidixic acid (30 µg)	Quinolones					+
Amikacin (30 µg)	Aminoglycosides			+	+	+
Gentamycin (10 µg)			+		+	
Kanamycin (30 µg)				+		+
Tobramycin (10 µg)					+	
Streptomycin (10 µg)			+			+
Erythromycin (15 µg)	Macrolides	+	+			
Clindamycin (2 µg)	Lincosamides		+			
Chloramphenicol (30 µg)	Phenicols	+	+	+		+
Tetracycline (30 µg)	Tetracyclines	+	+	+	+	+
Doxycycline (30 µg)			+			
Minocycline (30 µg)			+			
Nitrofurantoin (300 µg)	Nitrofurans		+			+
Rifampin (5 µg)	Rifamycins		+			
Trimethoprim-sulfamethoxazole (25 µg)	Sulfonamides		+	+	+	+
Linezolid (30 µg)	Oxazolidinones		+			
Vancomycin (30 µg)	Glycopeptides	+				
Vancomycin hydrochloride (Combi-Blocks, USA)		+				
Colistin sulfate (Sigma, Germany)	Polymyxins			+	+	+

### 2.4. Biofilm formation

Biofilm formation was assayed for *K. pneumoniae*, *A. baumannii*, and *E. coli* by the semi-quantitative method in a 96-well microtiter plate as described by Hu et al (2016) [34]. Briefly, cultures were inoculated in LB broth and adjusted to an optical density of 0.1 at 600 nm. For each isolate, three wells were filled with 200 µL of bacterial suspension. The negative control wells were filled with sterile LB broth. Plates were incubated for 24 h at 37 °C. Unattached bacteria were removed by three gentle washes with phosphate-buffered saline, air-dried, and stained with 0.1% crystal violet solution for 20 min. Crystal violet was then discarded, and the plate was washed four times with tap water and then air-dried. Ethanol (95% v/v) was added to solubilize the crystal violet dye, and plates were scanned at 570 nm to determine the optical density (OD) of the stained biofilms.

For *Enterococcus* spp., the modified protocol described by Khalil et al. (2023) [35] was used. Briefly, cultures were inoculated in trypticase soy broth (TSB; Oxoid, UK) supplemented with 1% glucose and adjusted to an optical density of 0.1 at 600 nm. For each isolate, three wells were filled with 200 µL of bacterial suspension. The negative control wells were filled with sterile TSB broth supplemented with 1% glucose. Plates were incubated for 24 h at 37 °C. Unattached bacteria were removed by three gentle washes with phosphate-buffered saline. After that, heat fixation at 60 °C was performed for 20 min, and plates were stained with 175 µL of 0.1% crystal violet for 20 min. Crystal violet was then discarded, and the plates were washed four times with sterile distilled water and air dried. Ethanol (95% v/v) was then added to solubilize the crystal violet dye, and plates were scanned at 570 nm to determine the OD of the stained biofilms.

For *S. aureus*, the modified protocol described by Singh et al. (2017) [36] was used. Briefly, cultures were inoculated in brain heart infusion broth (BHI; Oxoid, UK) supplemented with 222.2 mM glucose, 116.9 mM sucrose, and 1000 mM NaCl and adjusted to an optical density of 0.1 at 600 nm. Then, 10 µL was added from this suspension into a well containing 190 µL of BHI broth, and the plate was incubated for 24 h at 37 °C. Unattached bacteria were removed by three gentle washes with phosphate-buffered saline. After that, heat fixation at 60 °C was performed for 20 min, and plates were stained with 175 µL of 0.5% crystal violet for 5 min. Crystal violet was then discarded, and plates were washed four times with tap water and air dried. An ethanol–acetone mixture (80:20) was added for 30 min to solubilize the crystal violet dye, and plates were scanned at 550 nm to determine the OD of the stained biofilms.

For each isolate, the biofilm assay was performed in three independent experiments. The optical densities (ODs) of the three independent plates were compared with the cutoff OD (ODc). ODc = ODavg of the negative control + 3 × SD of ODs of the negative control. Each isolate was classified as follows: non-biofilm producer: OD ≤ ODc; weak biofilm producer: ODc < OD ≤ 2 × ODc; moderate biofilm producer: 2 × ODc < OD ≤ 4 × ODc; strong biofilm producer: OD > 4 × ODc, as described by Stepanović et al. (2000) [37].

## 3. Results

### 3.1. Isolation and identification of ESKAPE bacteria

Different selective media were used to isolate six major pathogenic and nosocomial bacteria belonging to the ESKAPE group from a total of 820 swab samples. Overall, bacterial isolates belonging to the targeted groups were recovered from 90 (11.0%) of the samples. *S. aureus* was the most frequently isolated species, with 39 isolates recovered, representing a prevalence of 4.8%. Of these isolates, 17 (43.6% of *S. aureus*; 2.1% of total samples) were confirmed as methicillin-resistant *S. aureus* (MRSA). *A. baumannii* was recovered from 17 samples (2.1% prevalence). Sixteen samples (1.95%) harbored *E coli*, none of which were identified as serotype O157:H7. *K. pneumoniae* was detected in 12 samples (1.5%). The lowest prevalence was observed for *Enterococcus spp*., found in only 6 samples (0.73%); further identification showed four of these were *E. faecalis* (0.49% overall prevalence), and two were *E. faecium* (0.24% overall prevalence). Notably, *P. aeruginosa* was not isolated from any of the 820 samples tested (Table 3).

**Table 3.** Prevalence of bacterial species isolated in this study.

Bacterial species/group	Number of isolates	Prevalence (820 samples)	Notes
*S. aureus*	39	4.8%	
MRSA	17	2.1%	43.6% of *S. aureus* isolates
*A. baumannii*	17	2.1%	
*E. coli*	16	1.95%	None identified as O157:H7
*K. pneumoniae*	12	1.5%	
*Enterococcus* spp.	6	0.73%	
*E. faecalis*	4	0.49%	
*E. faecium*	2	0.24%	
*P. aeruginosa*	0	0.0%	
Total isolates found	90	11.0%	Represents % of samples yielding ≥1 isolate

### 3.2. Antibiotic susceptibility testing

**Figure 1.**
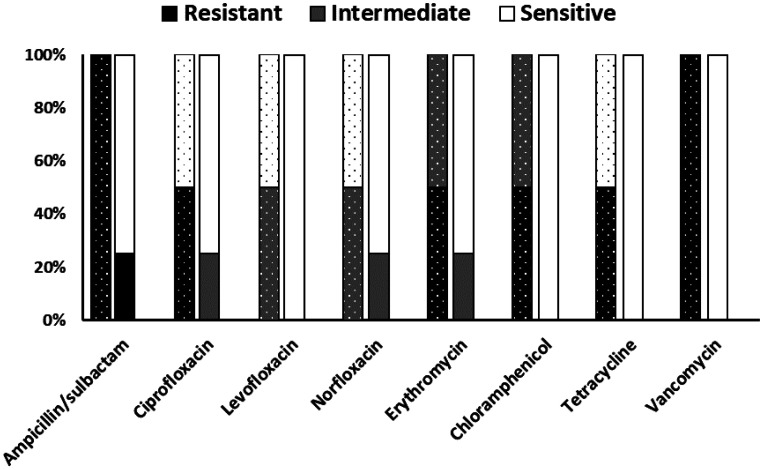
In vitro antibiotic susceptibility results for *Enterococcus faecalis* and *Enterococcus faecium* isolates (*N* = 6). Stacked bars show the percentage of isolates exhibiting resistance (black portion), intermediate susceptibility (grey portion), and sensitivity (white portion) to each antimicrobial agent tested, based on CLSI breakpoints. *E. faecalis*: Solid color-filled bars; *E. faecium*: Dot-filled bars.

High antibiotic susceptibility was observed among *E. faecalis* isolates (Figure 1). All tested isolates were susceptible to vancomycin, chloramphenicol, tetracycline, and levofloxacin. Resistance to the ampicillin/sulbactam combination was detected in one isolate. Moreover, all isolates were sensitive to vancomycin when tested using the MIC test. On the other hand, *E. faecium* showed a slightly different pattern of susceptibility toward tested antibiotics (Figure 1). All isolates were resistant to ampicillin/sulbactam and vancomycin, while half of the isolates were resistant to ciprofloxacin, erythromycin, chloramphenicol, and tetracycline. Besides, all isolates were resistant to vancomycin.

**Figure 2.**
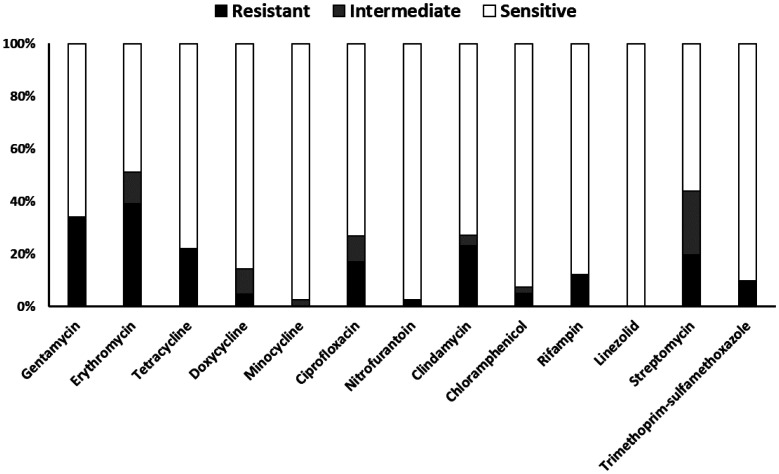
In vitro antibiotic susceptibility results for *Staphylococcus aureus* isolates (*N* = 39). Stacked bars show the percentage of isolates exhibiting resistance (black portion), intermediate susceptibility (grey portion), and sensitivity (white portion) to each antimicrobial agent tested, based on CLSI breakpoints.

*S. aureus* isolates displayed variable susceptibility to the tested antibiotics (Figure 2). All isolates were sensitive to linezolid, and high sensitivity rates (>90%) were also observed for minocycline, nitrofurantoin, trimethoprim-sulfamethoxazole, and chloramphenicol. Conversely, significant resistance was most prominent against erythromycin (39%) and gentamicin (34%). Other agents like tetracycline, clindamycin, and rifampin showed moderate resistance (12%–23%), while 25% of the isolates exhibited intermediate sensitivity against streptomycin. The MIC testing showed that 43.6% of isolates were resistant to cefoxitin. Overall, while several agents remain highly effective, considerable resistance exists toward commonly used drugs like erythromycin and gentamicin.

**Figure 3.**
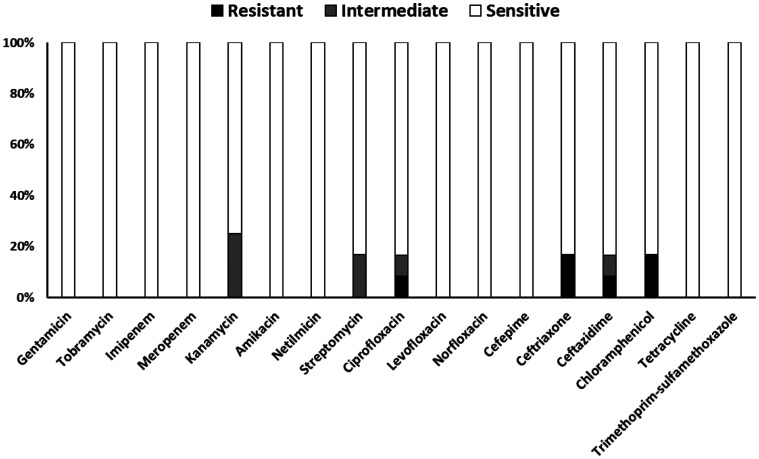
In vitro antibiotic susceptibility results for *Klebsiella pneumoniae* isolates (*N* = 12). Stacked bars show the percentage of isolates exhibiting resistance (black portion), intermediate susceptibility (grey portion), and sensitivity (white portion) to each antimicrobial agent tested, based on CLSI breakpoints.

The in vitro antibiotic susceptibility patterns of the *K. pneumoniae* indicate high susceptibility rates to the majority of the tested agents (Figure 3). Complete susceptibility was observed among all isolates for gentamicin, tobramycin, amikacin, and netilmicin, the carbapenems imipenem and meropenem, levofloxacin, norfloxacin, cefepime, tetracycline, and trimethoprim-sulfamethoxazole. For kanamycin, 75% of isolates were categorized as susceptible and 25% as intermediate, with no resistance detected. Similarly, for streptomycin, 83% of isolates were susceptible, and 17% were intermediate. Resistance was observed for four antibiotics, namely ciprofloxacin (8%), ceftazidime (8%), ceftriaxone (17%), and chloramphenicol (17%).

**Figure 4.**
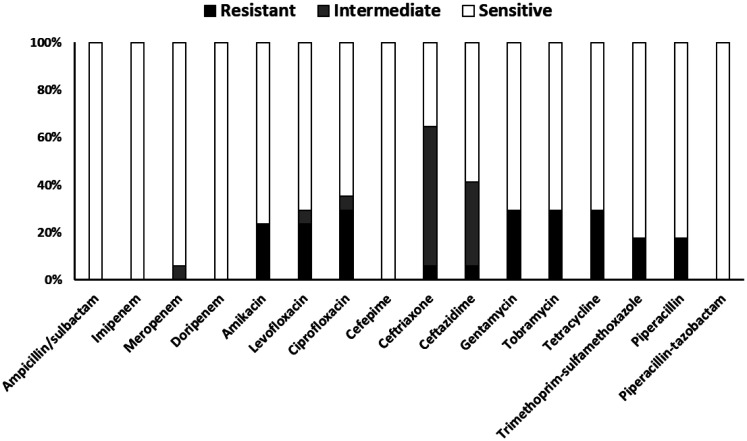
In vitro antibiotic susceptibility results for *Acinetobacter baumannii* isolates (*N* = 16). Stacked bars show the percentage of isolates exhibiting resistance (black portion), intermediate susceptibility (grey portion), and sensitivity (white portion) to each antimicrobial agent tested, based on CLSI breakpoints.

Figure 4 shows the susceptibility profiles of *A. baumannii* isolates. Complete susceptibility (100%) was observed for several antibiotics, including ampicillin/sulbactam, imipenem, doripenem, cefepime, and piperacillin-tazobactam. Among the carbapenems, meropenem showed susceptibility in 94% of isolates, with 6% classified as intermediate. The highest resistance rate recorded was 29.4%, observed equally against ciprofloxacin, gentamicin, tobramycin, and tetracycline. Ceftriaxone and ceftazidime showed unique patterns with low resistance but high intermediate rates of 60% and 38%, respectively. Both trimethoprim-sulfamethoxazole and piperacillin (tested without tazobactam) showed resistance in 18% of isolates, with the remaining 82% being susceptible in each case.

**Figure 5.**
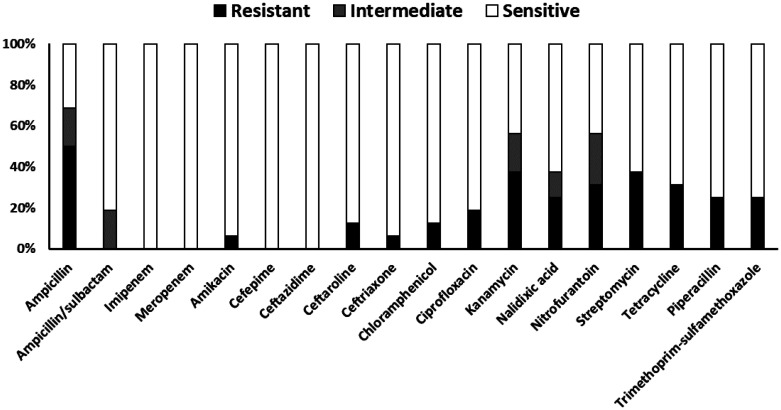
In vitro antibiotic susceptibility results for *Escherichia coli* isolates (*N* = 17). Stacked bars show the percentage of isolates exhibiting resistance (black portion), intermediate susceptibility (grey portion), and sensitivity (white portion) to each antimicrobial agent tested, based on CLSI breakpoints.

All tested *E. coli* isolates were sensitive to imipenem, meropenem, cefepime, and ceftazidime (Figure 5). In contrast, the highest rate of resistance was observed against ampicillin (50%), followed by kanamycin and streptomycin, both with resistance rates of 38%. High resistance rates exceeding 30% were also noted for nitrofurantoin and tetracycline. Moderate resistance rates of approximately 25% were observed for nalidixic acid, piperacillin, and trimethoprim-sulfamethoxazole. Lower resistance frequencies were recorded for ceftaroline (13%), chloramphenicol (13%), ceftriaxone (6%), and amikacin (6%).

### 3.3. Biofilm formation assay

**Figure 6.**
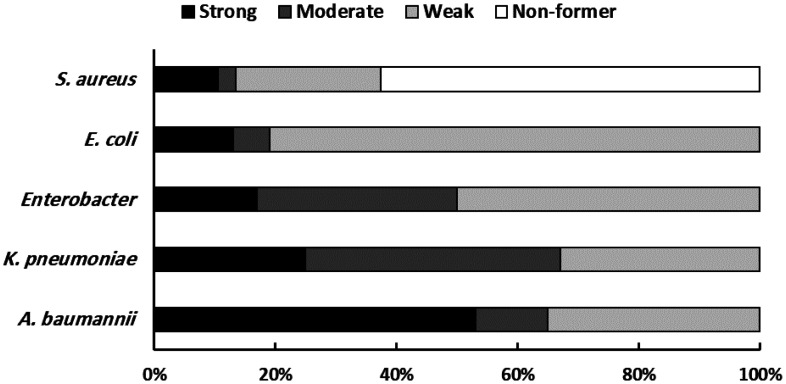
Biofilm formation phenotype for all recovered ESKAPE bacterial isolates in this study.

The biofilm formation capabilities of the recovered ESKAPE pathogen isolates were quantitatively assessed and categorized (Figure 6). Examination of the four *Enterococcus faecalis* isolates revealed no strong biofilm producers; one isolate (25%) was classified as a moderate biofilm former, while the remaining three (75%) demonstrated weak biofilm formation. Both of the *Enterococcus faecium* isolates were capable of biofilm production, with one exhibiting a strong phenotype and the other (50%) a moderate phenotype. Among the 39 *S. aureus* isolates, biofilm formation ability varied considerably, as nine isolates (23.1%) were strong biofilm producers, one isolate (2.6%) was moderate, and eight (20.5%) were weak producers. Notably, the majority of *S. aureus* isolates, 21 (53.8%), were categorized as non-formers of biofilm under the assay conditions. All 12 *K. pneumoniae* isolates were able to form biofilms, with three (25%) demonstrating strong formation, five (41.7%) moderate formation, and four (33.3%) weak formation. Similarly, all 17 *A. baumannii* isolates produced biofilms; nine (53%) were strong biofilm formers, representing the majority for this species, while two (11.8%) were moderate and six (35.3%) were weak formers. Lastly, biofilm production was also detected in all 16 *Escherichia coli* isolates, with the weak phenotype being predominant: 13 isolates (81.25%) were weak biofilm formers, compared to two (12.5%) strong formers and one (6.25%) moderate former. No non-biofilm forming isolates were identified among the *E. faecalis*, *E. faecium*, *K. pneumoniae*, *A. baumannii*, or *E. coli* groups tested.

## 4. Discussion

This study investigated the prevalence of antibiotic susceptibility and biofilm formation capacity of ESKAPE pathogens recovered from shopping cart handles in different cities in Jordan. Of the 820 swab samples collected, *S. aureus* was the most frequently isolated bacterium, present on 4.8% of sampled handles. While this prevalence is lower than the 14%, 17.3%, and 35% reported in studies by Al-Ghamdi et al. (2011), Patel et al. (2023), and Premanath et al. (2025), respectively, it confirms shopping carts as a potential reservoir [17],[23],[38]. Most of the recovered *S. aureus* isolates in this study were antibiotic-sensitive and biofilm non-formers. These phenotypes might indicate the background presence of typical commensal *S. aureus* strains commonly inhabiting human skin and nasopharyngeal passages [39]–[41]. The identification of MRSA strains among the isolates, which were recovered from 2.1% of sampled handles, is of significant clinical and public health interest. The MRSA isolates might have been shed from human users of the carts, either directly from the hands of individuals colonized or infected with MRSA, or indirectly by shedding of skin carrying the bacterium. The result aligns with the findings of Domon et al. (2016), who linked the infrequent detection of MRSA on such surfaces to its lower prevalence (~2.1%) compared to MSSA (~29.4%) within the healthy community [42].

*A. baumannii* was the second most prevalent among the recovered isolates, with a prevalence of 2.1%, comparable to previous local reports [43]. This result is far lower than the results reported by Premanath et al. (2025) (16%) [38], who reported that *A. baumannii* prevalence was third after *S. aureus* and *K. pneumonia*. Most of the *A. baumannii* isolates were highly sensitive to the tested antibiotics but were strong biofilm formers. This strong biofilm capacity aligns with prior findings [43] and one key characteristic contributing to *A. baumannii's* environmental persistence on diverse surfaces, potentially enhanced by interactions within polymicrobial communities [44]–[46]. While the observed antibiotic susceptibility contrasts with the global prevalence of multidrug-resistant (MDR) and extensively drug-resistant (XDR) clinical isolates [47]–[49], it might indicate that the *A. baumannii* isolates recovered in this study are community-associated strains [50]. Shopping carts in hypermarkets and produce markets are used to transport food such as fresh produce and meat. Therefore, *A. baumannii* isolates could be transmitted from the transported food, as it has been isolated extensively from food in several studies [51]–[56]. Nevertheless, the presence of strongly biofilm-forming *A. baumannii* on high-contact community surfaces such as cart handles is significant, given the ability of this pathogen to adapt to different environments, as well as acquire and disseminate resistance determinants.

The coliforms *E. coli* and *K. pneumonia* were isolated from shopping cart at rates of 1.95% and 1.5%, respectively. Most of the isolates were antibiotic-sensitive and weak biofilm formers. Their presence indicates fecal contamination, which may have occurred through environmental sources like animals and bird droppings when carts are parked outdoors [57]. Also, inadequate hand hygiene of users or staff, or insufficient routine cleaning of heavily used carts, may contribute to this type of contamination, as shown in Al-Ghamdi et al. (2011). Indeed, many studies reported the presence of *E. coli* and *K. pneumonia* on shopping cart handles in several studies, which is consistent with our findings [15],[18],[19],[21]–[23],[57]. The source of these pathogens might be from contacts between shopping carts and vegetables and fruits, which might be contaminated with animal feces.

*Enterococcus spp*. showed the lowest prevalence in our study (0.73%), contrasting with variable rates reported by other studies, such as 29.2% by Ashgar and El-Said (2012) and 1.7% by Carrascosa et al. (2019) [18],[19]. Interestingly, *E. faecium* isolates were highly resistant to the tested antibiotics, especially vancomycin. The presence of vancomycin-resistant *Enterococcus* VRE on frequently touched surfaces like cart handles poses a significant public health concern, given VRE's association with clinical outbreaks and mortality [58]. The circulation of VRE on cart handles might lead to the transfer of resistance determinants to other pathogens like *S. aureus*, especially MRSA [59].

This study provides significant insight into the contamination of high-touch community surfaces in Jordan, successfully identifying clinically relevant pathogens. This study has a limitation related to sampling design, which must be taken into consideration when interpreting the study findings. Our results provide a snapshot rather than seasonally or geographically generalizable estimates, due to the cross-sectional, single-country sampling strategy adopted in our study. Another limitation is the use of swab-recovery and culture-based approach, which may under-detect low-prevalence or fastidious organisms. In addition, bacterial load as CFU per area was not quantified, which could enable us to compare contamination levels across sites and time. Lastly, the study did not include whole-genome sequencing (WGS) or multi-locus sequence typing (MLST) techniques. Therefore, we cannot determine the genetic relatedness of the isolates or trace the source of clinically significant strains like MRSA and VRE.

## 5. Conclusions

This research demonstrates that shopping cart handles in Jordan are contaminated with various ESKAPE pathogens, exhibiting diverse antibiotic resistance and biofilm-forming capabilities. The presence of virulent and resistant strains, such as MRSA and VRE, on these high-contact surfaces underscores their role as potential vectors for bacterial transmission within the community. Our findings highlight the critical need for implementing strict, regular sanitation protocols for shopping carts to reduce public health risks. This can be done by periodically disinfecting shopping cart handles with an effective antiseptic. In addition, supermarkets should provide hygienic solutions near the cart area where users could disinfect their hands upon finishing their cart use. In addition, periodic surveillance for pathogens and enhanced public awareness initiatives, such as educating supermarket workers on the dangers posed by shopping carts and on proper cleaning practices, are highly recommended.

## Use of AI tools declaration

The authors declare they have not used Artificial Intelligence (AI) tools in the creation of this article.
